# Cod-Liver Oil in Strumous Affections

**Published:** 1846-12

**Authors:** 


*Topical Employment of Cod-Liver Oil, in the Treatment of certain Strumous Affections.*—By Dr. Brefeld. In cases of tumefaction of the lymphatic glands of the neck, axilla, and groin, this physician prescribes, with success, the employment of cod-liver oil in frictions to the inflamed and painful parts. He says that he has observed that this topical medication becomes useless, when it is used with tumors which are the consequence of variola, scarlatina, or rubeola. In the case of scrofulous ulcers, consecutive to inflammation and suppuration of the lymphatic ganglions, he employs the following ointment:—Oil of cod-livers, 15 parts. Liquor of subacet. of lead, 8 parts. Yolk of egg, 12 parts. Make into a homogenous ointment.

We can, in the preparation of this ointment, replace the yolk of egg by an equal quantity of lard. This ointment may be applied to the ulcers, lightly, by means of a feather. In scrofulous ophthalmia, M. Brefeld smears, twice or three times a day, the margins of the eyelids, with pure cod-liver oil, which he applies by means of a camel's hair brush, or a feather. In scrofulous peritonitis, this practitioner rubs the cod-liver oil on the surface of the abdomen; and when the latter is painful, he recommends to warm the oil.—*Jour. de Phar.—Dub. Hos. Gaz.—Ibid.*

